# Assessing Symptom Burden and Depression in Subjects With Chronic Respiratory Insufficiency

**DOI:** 10.1177/08258597211049592

**Published:** 2021-11-29

**Authors:** Heidi A. Rantala, Sirpa Leivo-Korpela, Lauri Lehtimäki, Juho T. Lehto

**Affiliations:** 1 Department of Respiratory Medicine, Tampere University Hospital, Tampere, Finland; 2 Faculty of Medicine and Health Technology, Tampere University, Tampere, Finland; 3 Allergy Centre, Tampere University Hospital, Tampere, Finland; 4 Department of Oncology, Palliative Care Unit, Tampere University Hospital, Tampere, Finland

**Keywords:** chronic respiratory insufficiency, Edmonton symptom assessment system, depression, symptoms, chronic obstructive pulmonary disease

## Abstract

**Objectives:** Patients with chronic respiratory insufficiency suffer from advanced disease, but their overall symptom burden is poorly described. We evaluated the symptoms and screening of depression in subjects with chronic respiratory insufficiency by using the Edmonton symptom assessment system (ESAS). **Methods:** In this retrospective study, 226 subjects with chronic respiratory insufficiency answered the ESAS questionnaire measuring symptoms on a scale from 0 (no symptoms) to 10 (worst possible symptom), and the depression scale (DEPS) questionnaire, in which the cut-off point for depressive symptoms is 9. **Results:** The most severe symptoms measured with ESAS (median [interquartile range]) were shortness of breath 4.0 (1.0-7.0), dry mouth 3.0 (1.0-7.0), tiredness 3.0 (1.0-6.0), and pain on movement 3.0 (0.0-6.0). Subjects with a chronic obstructive pulmonary disease as a cause for chronic respiratory insufficiency had significantly higher scores for shortness of breath, dry mouth, and loss of appetite compared to others. Subjects with DEPS ≥9 reported significantly higher symptom scores in all ESAS categories than subjects with DEPS <9. The area under the receiver operating characteristic curve for ESAS depression score predicting DEPS ≥9 was 0.840 (*P* < .001). If the ESAS depression score was 0, there was an 89% probability of the DEPS being <9, and if the ESAS depression score was ≥4, there was an 89% probability of the DEPS being ≥9. The relation between ESAS depression score and DEPS was independent of subjects’ characteristics and other ESAS items. **Conclusions:** Subjects with chronic respiratory insufficiency suffer from a high symptom burden due to their advanced disease. The severity of symptoms increases with depression and 4 or more points in the depression question of ESAS should lead to a closer diagnostic evaluation of depression. Symptom-centered palliative care including psychosocial aspects should be early integrated into the treatment of respiratory insufficiency.

## Introduction

Chronic respiratory insufficiency and the need for noninvasive ventilation (NIV) or long-term oxygen therapy (LTOT) are often signs of advanced disease and poor prognosis. Patients with advanced respiratory disease typically have severe breathlessness, but some other symptoms are reported as well.^1–5^ Patients may suffer from pain, loss of energy, dry mouth, cough, depression, and anxiety in addition to dyspnea.^6–9^ Comorbidities further enhance the symptom burden in advanced respiratory diseases and significantly affect patients’ quality of life and survival.^10,11^ Guidelines and previous studies have recommended to systematically screen symptoms other than dyspnea in patients with advanced respiratory disease.^1,12,13^

Depression is common in diseases causing chronic respiratory insufficiency, such as chronic obstructive pulmonary disease (COPD) and interstitial lung disease.^3,9,14^ Depressive symptoms increase perceived symptom burden and impair quality of life.^3,4,15,16^ They have also been associated with a risk of hospitalization, emergency care use, and impaired prognosis.^14,17,18^ Therefore, systematic screening of depression in patients with advanced respiratory diseases and chronic respiratory insufficiency is warranted.

Patients with chronic respiratory insufficiency suffer from multimorbidity demanding medical attention. Their proper treatment requires knowledge of individual symptom burdens beyond breathlessness. Previous studies have focused on different advanced pulmonary diseases, but not on an unselected population of chronic respiratory insufficiency.^1,2,9,16^ Therefore, studies describing the overall symptom burden, prevalence of depression, or the relationship between depression and other symptoms in patients with chronic respiratory insufficiency are needed.

We aimed to describe the overall symptom burden in subjects with chronic respiratory insufficiency and to assess the association between depression and other symptoms. We also assessed how well a single question on depression as part of the Edmonton symptom assessment system (ESAS) reflects depressive symptoms measured by a more thorough depression scale (DEPS) questionnaire.

## Methods

All patients with chronic respiratory insufficiency who visited the respiratory insufficiency clinic of Tampere University Hospital from October 1, 2016, to October 31, 2017, were included in this retrospective study. Information on sex, age, weight, height, living conditions, smoking status, diagnoses, microspirometry results, scores of ESAS and DEPS, and the date of death (follow-up until December 31, 2018) were collected from the medical records. The disease-causing chronic respiratory insufficiency and the need for NIV or LTOT were defined as the primary disease, while all other diseases were considered as comorbidities. The Charlson comorbidity index was calculated for the subjects based on the number and severity of their comorbidities.^19,20^

### Questionnaires

In the respiratory insufficiency unit of Tampere University Hospital, patients are asked to complete the ESAS and DEPS in addition to the normal medical assessment. The ESAS was originally developed for assessing the symptoms of patients with advanced cancer in palliative care, but it is a commonly used method for assessing symptoms in patients with many advanced diseases.^21,22^ In the ESAS, different symptoms perceived on that day are measured on a numeric rating scale from 0 (no symptoms) to 10 (the worst possible symptoms).^23,24^ In our clinic, we use a modified version with 12 questions covering 11 symptoms (pain at rest, pain on movement, tiredness, shortness of breath, loss of appetite, nausea, dry mouth, constipation, depression, anxiety, and insomnia) and general well-being (0 for the best possible well-being and 10 for the worst possible well-being).

The DEPS is a validated, self-assessed screening tool for depression.^25^ The DEPS questionnaire consists of 10 questions and provides a total score varying from 1 to 30 points. The suggested cut-offs for depressive symptoms and clinical depression are ≥9 and ≥12, respectively.^26^ The cut-off level of 9 points has a high sensitivity to detect the possibility of depression and is therefore used as a threshold for further diagnostic evaluation of depression.^26–29^

### Statistical Analysis

The distributions were nonnormal based on Shapiro–Wilk test and, thus, nonparametric tests were used. Comparisons of different groups were performed by the Mann-Whitney *U* test or Kruskal–Wallis test for continuous variables and Pearson's chi-square test or Fisher's exact test for categorical variables. To evaluate the capacity of the ESAS depression score to predict DEPS ≥9, a receiver operating characteristic curve (ROC) analysis was performed. Additionally, the sensitivity, specificity, positive predictive value (PPV), and negative predictive value (NPV) of the values were calculated. To assess if the relation between depression and other symptoms is independent of other demographic factors, we conducted a logistic regression multivariate analysis. Statistical significance was set as *P *< .05. Analyses were performed with IBM SPSS Statistics version 26.0. (IBM Corp.).

### Ethical Consideration

This study was approved by the Regional Ethics Committee of Tampere University Hospital, Finland (approval code R15180 / December 1, 2015).

## Results

Altogether, 270 subjects with chronic hypoxemic or hypercapnic respiratory insufficiency visited the respiratory insufficiency clinic of Tampere University Hospital during the follow-up time. Of those, 226 subjects had completed the ESAS questionnaire. The reasons for missing ESAS results were as follows: inability to complete the questionnaire (*n* = 9), unwillingness to answer the questionnaire (*n* = 4), and technical or unknown reasons (*n* = 31). Of these 226 subjects with ESAS questionnaires, DEPS was available for 208 subjects. The reasons for missing DEPS were as follows: unwillingness to answer the questionnaire (*n* = 4) and technical or unknown reasons (*n* = 14).

The subject characteristics are shown in Table 1. The most common primary diseases causing the need for NIV or LTOT were COPD and obesity-hypoventilation syndrome (OHS). The treatment for respiratory insufficiency was NIV in 92 (40.7%) and LTOT in 85 (37.6%) of the subjects, while 21 (9.3%) of the subjects had both NIV and LTOT. Twenty-two subjects had only portable oxygen, and 1 subject was treated with continuous positive airway pressure (CPAP) due to OHS. Five subjects with respiratory insufficiency refused to use NIV or LTOT. Of the deceased subjects, 59.2% died during the first year after visiting the respiratory insufficiency clinic.

**Table 1.** Subject Characteristics in all Subjects and According to the Primary Diagnosis Causing Respiratory Insufficiency.

	All subjects (*n* = 226)	COPD (*n* = 104)	OHS (*n* = 61)	Others^a^ (*n* = 61)	*P*-value*
Gender, *n* (%)					
Male	130 (57.5)	69 (66.3)	31 (50.8)	30 (49.2)	.046
Female	96 (42.5)	35 (33.7)	30 (49.2)	31 (50.8)	
Age, median (IQR)	72.0 (65.0-79.0)	73.0 (68.3-79.0)	69.0 (61.0-78.0)	71.0 (60.5-80.0)	.009
BMI (kg/m^2^), median (IQR)^b^	30.0 (23.8-38.5)	24.6 (20.8-30.7)	42.3 (38.5-49.9)	28.8 (24.9-32.5)	<.001
Smoking status, *n* (%)					
Never-smoker	65 (28.8)	3 (2.9)	23 (37.7)	39 (63.9)	<.001
Ex-smoker	141 (62.4)	94 (90.4)	27 (44.3)	20 (32.8)	
Smoker	19 (8.4)	7 (6.7)	11 (18.0)	1 (1.6)	
Unknown	1 (0.4)	0 (0.0)	0 (0.0)	1 (1.6)	
FEV_1_, liters, median (IQR)^c^	1.16 (0.71-1.67)	0.78 (0.57-1.18)	1.60 (1.20-1.95)	1.37 (0.96-1.80)	<.001
Charlson comorbidity index, median (IQR)	2.0 (1.0-3.0)	2.0 (0.0-2.0)	2.0 (1.0-3.0)	1.0 (0.0-2.5)	.032
Died before December 31, 2018, *n* (%)	71 (31.4)	44 (42.3)	7 (11.5)	20 (32.8)	<.001

Abbreviations: COPD, chronic obstructive pulmonary disease; OHS, obesity-hypoventilation syndrome; IQR, interquartile range; BMI, body mass index; FEV_1_, forced vital capacity in 1 s.

*P-value between the subjects with COPD, OHS, and others.

a Others consisting of neurological diseases (*n* = 14), thoracic deformity (*n* = 14), interstitial lung diseases (*n* = 13), heart diseases (*n* = 12), sleep apnea (*n* = 2), and others (*n* = 6).

b Data were missing in 1 subject: *confined to bed*.

c Data were missing in 7 subjects*: lack of cooperation (3), no respiratory disease (4).*

Symptoms measured by ESAS in the whole group and according to the primary disease are shown in Table 2. In addition to shortness of breath, the most noticeable symptoms were dry mouth (3.0), pain on movement, and tiredness. Subjects with COPD reported more severe shortness of breath, dry mouth, and loss of appetite compared to other groups. Symptom scores for pain at rest were missing in 9 subjects and in other categories in 3 to 6 subjects.

**Table 2.** Scores and Prevalence of Symptoms Measured by the Modified ESAS Questionnaire According to the Primary Diagnosis Causing Respiratory Insufficiency.

	All subjects (*n* = 226)		COPD (*n* = 104)		OHS (*n* = 61)		Others*^b^* (*n* = 61)		
	Prevalence*^c^* (%)	Score, median (IQR)	Prevalence^c^ (%)	Score, median (IQR)	Prevalence^c^ (%)	Score, median (IQR)	Prevalence^c^ (%)	Score, median (IQR)	*P*-value*
*Symptoms*									
* *Pain at rest	54.4	1.0 (0.0-3.0)	50.5	1.0 (0.0-3.0)	62.5	2.0 (0.0-4.0)	52.5	1.0 (0.0-2.0)	.15
* *Pain on movement	71.8	3.0 (0.0-6.0)	70.3	2.0 (0.0-5.5)	72.9	4.0 (0.0-7.0)	73.3	2.5 (0.0-4.0)	.29
* *Tiredness	81.4	3.0 (1.0-6.0)	86.1	3.0 (2.0-6.0)	75.9	2.0 (0.8-6.0)	78.7	3.0 (1.0-5.0)	.18
* *Shortness of breath	84.2	4.0 (2.0-7.0)	95.1	6.0 (3.0-8.0)	74.1	3.0 (0.0-5.0)	75.0	3.0 (0.3-5.0)	<.001
* *Loss of appetite	49.5	0.0 (0.0-3.0)	62.1	1.0 (0.0-4.0)	34.5	0.0 (0.0-2.0)	42.6	0.0 (0.0-2.0)	.004
* *Nausea	29.5	0.0 (0.0-1.0)	33.0	0.0 (0.0-1.0)	24.6	0.0 (0.0-0.5)	28.3	0.0 (0.0-1.0)	.60
* *Dry mouth	80.3	3.0 (1.0-7.0)	87.5	5.0 (2.0-7.0)	67.8	2.0 (0.0-6.0)	80.0	3.0 (1.0-7.0)	.01
* *Constipation	51.1	1.0 (0.0-3.0)	53.4	1.0 (0.0-4.0)	47.4	0.0 (0.0-2.0)	50.0	1.0 (0.0-3.0)	.47
* *Depression	54.8	1.0 (0.0-3.5)	56.9	1.0 (0.0-4.0)	51.7	1.0 (0.0-3.3)	54.1	1.0 (0.0-3.0)	.91
* *Anxiety	52.5	1.0 (0.0-3.0)	55.9	1.0 (0.0-4.0)	46.6	0.0 (0.0-3.0)	52.5	1.0 (0.0-3.0)	.50
* *Insomnia	64.0	2.0 (0.0-4.0)	70.6	2.0 (0.0-4.0)	54.2	1.0 (0.0-5.0)	62.3	1.0 (0.0-3.0)	.40
* *Well-being		4.0 (2.0-5.0)		4.0 (2.5-5.0)		3.0 (1.8-5.3)		3.0 (2.0-5.0)	.20

Abbreviations: ESAS, Edmonton symptom assessment system; COPD, chronic obstructive pulmonary disease; OHS, obesity-hypoventilation syndrome.

*P-value for the difference in ESAS scores between the subjects with COPD, OHS, and others.

b Others consisting of neurological diseases (*n* = 14), thoracic deformity (*n* = 14), interstitial lung diseases (*n* = 13), heart diseases (*n* = 12), sleep apnea (*n* = 2), and others (*n* = 6).

c Prevalence is defined as a proportion of subjects with an ESAS score ≥1.

Of the subjects who completed the DEPS questionnaire, 81 (38.9%) scored ≥9 points reaching the cut-off for depressive symptoms. The symptom severities measured by ESAS in the subjects with <9 and ≥9 points in DEPS are shown in Table 3. The proportion of subjects having DEPS ≥9 points did not significantly differ between subjects with different primary diseases (41.7% in COPD, 38.2% in OHS, and 35.1% in other diseases, *P* = .72). All the symptoms were significantly more severe in subjects with DEPS ≥9 compared to those with DEPS <9.

**Table 3.** Scores and Prevalence of Symptoms Measured by the Modified ESAS Questionnaire According to DEPS Category.

	DEPS <9 (***n*** = 127)		DEPS ≥9 (***n*** = 81)		
	Prevalence**^a^** (%)	Score, median (IQR)	Prevalence^a^ (%)	Score, median (IQR)	***P***-value*
Symptoms^b^					
Pain at rest	50.8	1.0 (0.0-3.0)	61.0	2.0 (0.0-4.5)	.02
Pain on movement	66.9	2.0 (0.0-4.0)	84.6	4.5 (1.0-7.0)	.001
Tiredness	73.4	2.0 (0.0-4.0)	97.5	5.0 (3.0-7.0)	<.001
Shortness of breath	76.8	3.0 (1.0-5.0)	94.9	6.0 (3.0-8.0)	<.001
Loss of appetite	39.2	0.0 (0.0-2.0)	68.4	2.0 (0.0-5.0)	<.001
Nausea	19.2	0.0 (0.0-0.0)	50.6	1.0 (0.0-3.0)	<.001
Dry mouth	78.0	3.0 (1.0-5.0)	85.9	6.0 (3.0-8.0)	<.001
Constipation	41.6	0.0 (0.0-2.5)	67.9	2.0 (0.0-5.0)	<.001
Depression	37.6	0.0 (0.0-2.0)	87.3	4.0 (2.0-7.0)	<.001
Anxiety	33.6	0.0 (0.0-1.0)	86.1	4.0 (2.0-7.0)	<.001
Insomnia	51.2	1.0 (0.0-2.0)	83.5	3.0 (1.0-6.0)	<.001
Well-being		3.0 (1.0-5.0)		5.0 (4.0-6.0)	<.001

Abbreviations: ESAS, Edmonton symptom assessment system; DEPS, depression scale; IQR, interquartile range.

*P-value for the difference in medians of DEPS <9 and ≥9.

a Prevalence is defined as a proportion of subjects with ESAS score ≥1.

b Data were missing in 18 subjects: *unwilling to answer DEPS questionnaire (4), technical or unknown reason (14).*

**Figure 1.**
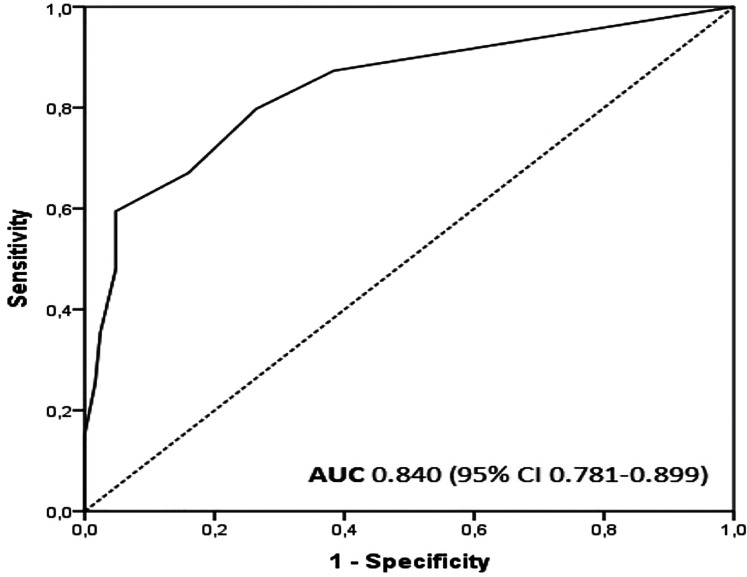
The ROC curve for the ESAS depression score to predict a DEPS score ≥9.

Abbreviations: ROC, receiver operating characteristic; ESAS, Edmonton symptom assessment system.

We evaluated the capacity of the ESAS depression score to predict whether DEPS is below 9 or at least 9 points by creating a ROC curve. The area under the ROC curve was 0.840 (*P *< .001) (Figure 1). The sensitivity, specificity, and positive and negative predictive values for ESAS scores to predict DEPS ≥9 are shown in Table 4. If the ESAS depression score was 0, there was an 89% probability that the subject would score below 9 points in DEPS. Similarly, if the subject reported at least 4 points in the ESAS depression score, there was an 89% probability for him/her to score at least 9 points in the DEPS.

**Table 4.** ESAS Depression Scores’ Capacity to Predict DEPS ≥9.

ESAS depression score	Sensitivity (%)	Specificity (%)	PPV (%)	NPV (%)	Accuracy (%)
≥1	87	62	59	89	72
≥2	80	74	66	85	76
≥3	67	84	73	80	77
≥4	59	95	89	79	81
≥5	48	95	86	74	77
≥6	35	98	90	71	74
≥7	25	98	91	68	70
≥8	15	100	100	65	67
≥9	4	100	100	62	63
10	3	100	100	62	62

Abbreviations: ESAS, Edmonton symptom assessment system; DEPS, depression scale; PPV, positive predictive value; NPV, negative predictive value.

DEPS was filled in 208 subjects.

To assess if the ESAS depression score is associated with DEPS independently of other ESAS questions and demographic details of subjects, we conducted a logistic regression model with DEPS ≥9 points as the dependent variable and gender, age, use of NIV, and use of LTOT and ESAS depression score as explanatory variables. In this model, only the ESAS depression score was independently associated with DEPS being ≥9 points. We then 1 by 1 added other ESAS variables to the model and only ESAS well-being and insomnia scores separately were associated with DEPS being ≥9 points, but they did not affect the significance of ESAS depression score in predicting DEPS ≥9 points. Further, they were not associated with DEPS being ≥9 points, if included both in the model (Supplemental Table 1). Other ESAS items and other demographic details were not significantly associated with DEPS ≥9 points and did not affect the relation between ESAS depression score and DEPS.

## Discussion

We showed a high symptom burden in subjects with chronic respiratory insufficiency. In addition to impaired well-being, the most severe symptoms were shortness of breath, pain on movement, tiredness, and dry mouth. Shortness of breath, dry mouth, and loss of appetite were more severe in subjects with COPD compared to other subjects. Subjects with depressive symptoms measured with the DEPS questionnaire had higher scores in all ESAS categories than those without. Compared to the more thorough DEPS, the single question on depression as part of the ESAS seems to work as a reasonable screening tool for depressive symptoms.

In clinical practice, it would be reasonable to have 1 simple screening tool for both depression and other symptoms. According to the present study, the ESAS could serve as such a tool. We showed that if a subject scores 4 or more in the ESAS depression category, the probability of depressive symptoms according to the DEPS is high. This same cut-off level has also been shown to be useful in detecting clinical depression defined by standardized questionnaires among patients with cancer.^30^ According to the logistic regression analysis, the relation between ESAS depression scores is not affected by subjects’ characteristics or other ESAS items. Based on the current results, we suggest that in patients scoring 0 on the ESAS depression question, there is probably no need for further evaluation of depression. Those scoring 1 to 3 would need to complete a specific depression questionnaire (eg, the DEPS), while those scoring 4 or more could be referred to clinical evaluation of depression. These cut-offs for clinical decision-making should be tested in further prospective studies.

In the present study, the subjects with chronic respiratory insufficiency, especially those with COPD, suffered from a high symptom burden measured with a systematic ESAS questionnaire. In previous studies, the symptoms described have varied according to the study design or disease severity, but they all have reported dyspnea as the main symptom.^2,6,31^ Blinderman et al^6^ described symptoms in patients with advanced COPD, for whom they did not report LTOT or NIV usage, and the most prevalent symptoms in addition to dyspnea were fatigue, xerostomia, coughing, pain, and anxiety. However, dry mouth was more prevalent in our subjects with chronic respiratory insufficiency than in the Blinderman study.^6^ In a recent study by Gainza-Miranda et al,^2^ the highest ESAS scores were found in dyspnea and loss of well-being in 60 patients with severe COPD and LTOT with or without NIV. This is in line with our study, but anxiety and depression were even more severe in the study by Gainza-Miranda et al,^2^ compared to the subjects with COPD in our study. In a study by Walke et al,^31^ patients with severe COPD reported shortness of breath, anxiety, and physical discomfort even more frequently than patients with cancer or chronic heart failure. There are only a few studies concerning symptoms on top of sleepiness in patients with OHS. In a small study evaluating 10 patients with OHS, Baris et al^16^ reported depression and anxiety in all patients. In the present study, the number of subjects with a primary diagnosis other than COPD or OHS was too small for any disease-specific conclusions. However, these subjects also suffered from multiple symptoms, and it is therefore essential to screen symptoms comprehensively in all patients with chronic respiratory insufficiency and to integrate symptom-centered palliative care into the treatment.

In the current study, 39% of the subjects with a completed DEPS questionnaire reached the threshold of 9 points for depressive symptoms without a significant difference between the subject groups. Our result is in line with a previous study in a similar patient population showing a prevalence of depressive symptoms of 34%.^29^ In a review by Smith and Wrobel,^15^ the prevalence of depression varied from 8% to 80% in patients with COPD, exceeding the prevalence found in the general population. Even in mild COPD, depression is reported in ∼15% of the patients. Lewis et al^32^ found no difference in the prevalence of depression between patients with COPD with or without LTOT as in both patient groups, the prevalence of depression was ∼15%. The prevalence of depression in patients with interstitial lung disease varies from 10% to 49%.^3,9,33^ In contrast to COPD and interstitial lung disease, there are only a few studies concerning depression in OHS.^16,34^

Higher scores on the DEPS questionnaire were associated with greater symptom scores in all ESAS categories in our subjects. Similar results have been reported among patients with advanced cancer.^35^ In COPD, clinically significant depression has been associated with a greater level of dyspnea and with other symptoms of COPD, such as cough, wheezing, and sputum production.^36^ Severe pulmonary disease with severe dyspnea can restrict a patient's ability to leave home or take part in activities, leading to social exclusion and later on to depressive symptoms or clinical depression. Moreover, depressive patients are physically less active, have impaired quality of life, and may experience their symptoms more severely than others.^35,37^ In addition to higher symptom burden, depression, and depressive symptoms are associated with increased risk of exacerbations, longer hospital stays, more frequent emergency room visits, and shorter survival in COPD.^37,38^ Depression may also influence compliance to treatment, and different treatments may influence depression. In a previous study, noncompliant LTOT users with COPD had major depression more often than compliant LTOT users.^39^ However, depressive symptoms are shown to decrease after CPAP therapy in OHS and by pulmonary rehabilitation in COPD.^16,34,40^ Regardless of the etiology of the chronic respiratory insufficiency, depressive symptoms are associated with many clinically important variables and should trigger a comprehensive symptom evaluation and therapy.

### Strengths and Limitations

We presented a real-life study on an unselected sample of subjects with chronic respiratory insufficiency. This study offers practical information on the symptom burden and screening of depressive symptoms in these subjects. The subjects with chronic respiratory insufficiency are a heterogeneous group with different underlying diseases, of which we focused on the most common ones, COPD and OHS, while other subject groups were too small to make conclusions.

We studied the overall symptom burden in these subjects during their treatment. However, we did not have the results of the ESAS questionnaires at the initiation of LTOT or NIV in every subject, which prevented us from evaluating the change in symptoms during the therapy. The ESAS questionnaire used in these subjects did not include questions concerning cough or secretions, which might have been informative in subjects with respiratory disease. Some of the subjects had missing values in the ESAS and the DEPS due to the retrospective nature of the study. Although the proportion of missing questionnaires was limited, it is possible that subjects with depression or without any symptoms were more unwilling to answer, which might have influenced our results. The questionnaires were given to the subjects at the same time when arriving at the clinic, but as they were not asked to fill the forms out in a specific order, the random order may have affected the results. Depressive symptoms in the ESAS were compared with a validated thorough questionnaire for depression (ie, the DEPS). However, we could not compare the results of the ESAS depression score to a clinical diagnosis of depression made by a psychiatrist. The focus of this study was to screen depressive symptoms in all subjects visiting a pulmonary unit to find those needing closer examination of depression.

## Conclusion

Systematic assessment with the ESAS questionnaire revealed that subjects with chronic respiratory insufficiency, especially those with COPD, suffer from multiple symptoms beyond breathlessness. The single ESAS depression question seems to serve as a reasonable screening tool for depressive symptoms, which were associated with a higher prevalence and severity of other symptoms. We suggest that there is a need for systematic symptom screening as well as integrated palliative care with psychosocial support for patients with chronic respiratory insufficiency.

## Supplementary Material

Supplementary material
