# Supplementary material for: Assessing Symptom Burden and Depression in Subjects With Chronic Respiratory Insufficiency
Source: J Palliat Care. 2021 Nov 29;37(2):134–41. doi: 10.1177/08258597211049592 (PMC9109583; doi:10.1177/08258597211049592)
Supplement: Supplementary material [file sj-docx-1-pal-10.1177_08258597211049592.docx]

**Supplementary Table 1. Association of DEPS ≥ 9 points with other variables in logistic regression analysis**

|  | HR (95% CI) | | P-value |
| --- | --- | --- | --- |
|  |  |  |  |
| Gender  Age  Usage of NIV  Usage of LTOT  ESAS depression  ESAS well-being  ESAS insomnia | 1.0 (0.4-2.3)  1.0 (1.0-1.1)  1.5 (0.6-3.7)  1.1 (0.4-2.7)  1.7 (1.4-2.1)  1.2 (1.0-1.5)  1.2 (1.0-1.4) |  | 0.996  0.094  0.385  0.865  < 0.001  0.087  0.079 |

DEPS, Depression scale; NIV, noninvasive ventilation; LTOT, long-term oxygen therapy, ESAS, Edmonton Symptom Assessment System
